# A case report of a large right atrial myxoma: the role of virtual consultations and imaging during the COVID-19 pandemic

**DOI:** 10.1093/omcr/omac059

**Published:** 2022-06-23

**Authors:** Anju JJ Velvet, Vishal Parekh, Waqas Khan, Irfan Ahmed

**Affiliations:** Department of Cardiology, Royal Preston Hospital, Lancashire Teaching Hospitals NHS Foundation Trust, Preston, UK; Department of Cardiology, Royal Preston Hospital, Lancashire Teaching Hospitals NHS Foundation Trust, Preston, UK; Department of Cardiology, Royal Preston Hospital, Lancashire Teaching Hospitals NHS Foundation Trust, Preston, UK; Department of Cardiology, Royal Preston Hospital, Lancashire Teaching Hospitals NHS Foundation Trust, Preston, UK

## Abstract

We present a case report of a right atrial myxoma first diagnosed on a transthoracic echocardiogram after telephone consultations held in lieu of face-to-face consultations during the first wave of the COVID-19 pandemic. The echocardiogram was requested on the second telephone consultation 3 months after an initial presentation with a dry cough and fatigue due to new symptoms of palpitations and shortness of breath raising suspicion of heart failure. Virtual consultations continue to replace face-to-face consultations to avoid unnecessary exposure to COVID and reduce health care costs. This case report focuses on the importance of obtaining a systematic history, identifying red flags, referring to appropriate specialties and requesting the right investigations for early diagnosis and management of conditions with serious complications.

## INTRODUCTION

Myxomas are the most common primary cardiac tumours, but less common in the right atrium (RA). We present a case of a large RA myxoma which was detected first on a transthoracic echocardiogram (TTE) following telephone consultations in a patient presenting initially with a dry cough and subsequently with symptoms of heart failure during the first wave of the Covid-19 pandemic.

## CASE REPORT

**Figure 1:**
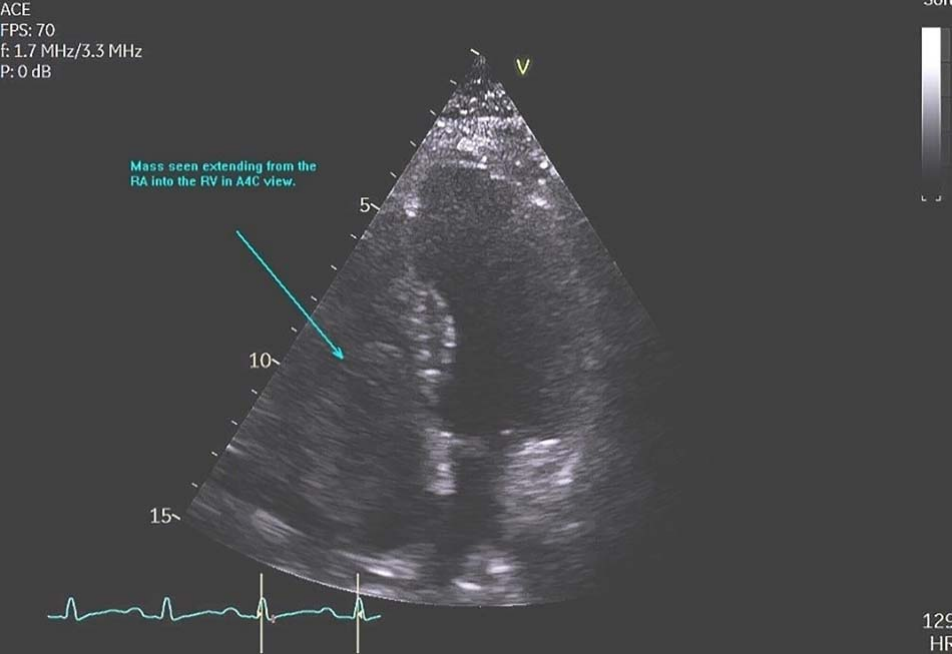
TTE showing RA myxoma seen extending into the RV in the apical four chamber view.

**Figure 2:**
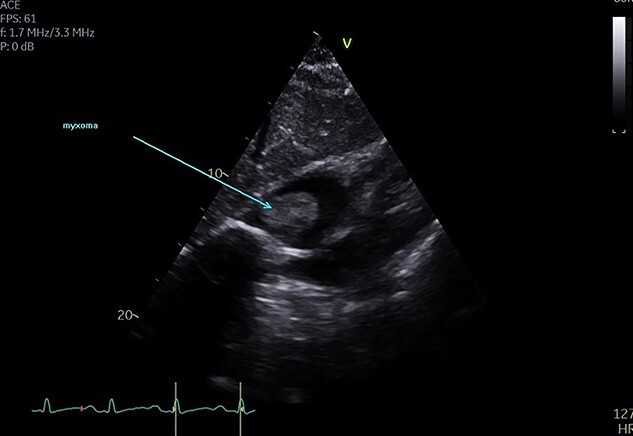
TTE showing RA myxoma in the subcostal view.

**Figure 3:**
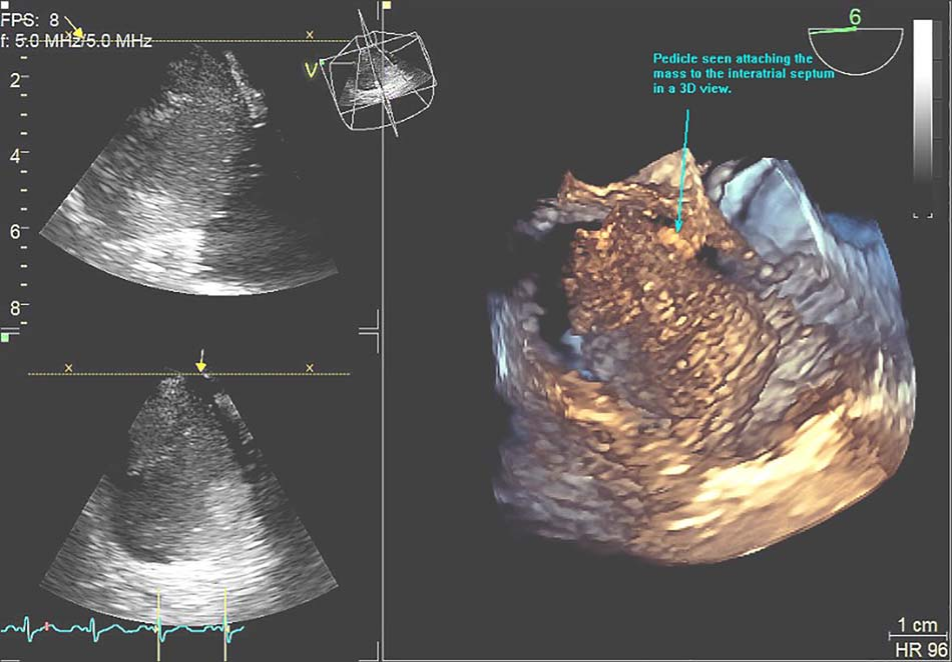
3D view of the RA myxoma with the pedicle attached to the IAS.

**Figure 4:**
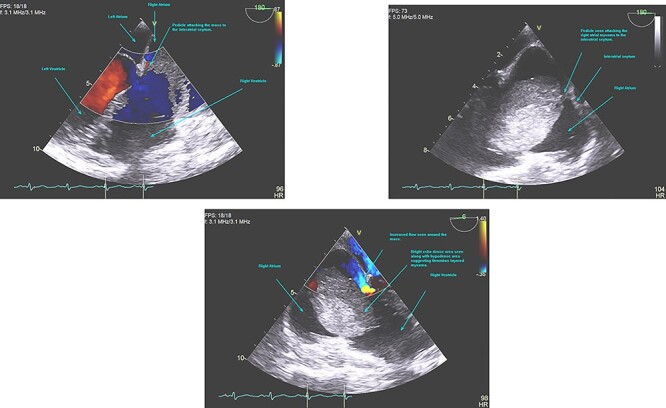
TOE showing myxoma in the RA with the pedicle attached to the IAS.

A 48-year-old Caucasian woman with a history of hypertension and acute myeloid leukaemia (2009) presented initially with a persistent dry cough and increased fatigue on a telephone consultation with her general practitioner (GP). This was arranged instead of a face-to-face consultation during the COVID-19 pandemic. She was first referred to the ENT surgeons as there was associated dysphagia; however, no abnormality was detected on examination, chest X-ray, nasal-endoscopy and barium swallow. About 3 months later she presented with new symptoms of palpitations, shortness of breath and chronic cough on a second telephone consultation with the GP. A TTE was arranged through the direct open access clinic due to concerns of heart failure. TTE showed a 12.5 cm^2^ mass in the RA, protruding into the RV through the tricuspid valve. RV size was normal but with impaired systolic function (Figs. 1, 2, 3). She was referred to cardiology and a transoesophageal echocardiogram (TOE) demonstrated a RA mass of 5.4 cm × 4.4 cm (Fig. 4) with a differential diagnosis (DD) of thrombus or myxoma. She was referred to a tertiary cardiology centre and had a CT scan, cardiac magnetic resonance imaging (CMRI) and a multidisciplinary team (MDT) meeting following which a diagnosis of atrial myxoma (AM) was made. She underwent a right mini-thoracotomy with AM excision. Histopathology reported a macroscopic appearance of a gelatinous, haemorrhagic mass weighing 38 g, measuring 55×45×25mm, with microscopic features of AM. She had a good postoperative recovery and was discharged 4 days postsurgery. Was seen in the cardiology follow-up clinic with a complete improvement of her symptoms and a TTE showing no recurrence.

## DISCUSSION

Virtual consultations have been in use for several decades even before the Covid-19 pandemic providing healthcare to patients in rural areas. Our patient initially presented with a persistent dry cough along with increased fatigue during the first wave of the COVID-19 pandemic. She was first referred to the ENT surgeons to look for any obstruction causing dysphagia and cough. No abnormality was detected on examination and investigations. She presented 3 months later, again on a telephone consultation with the GP but now her symptoms had progressed and were associated with palpitations and shortness of breath, raising concerns of heart failure. A TTE is then requested and a referral is made to cardiology. This demonstrates the importance of recognizing red flags from a systematic history on a virtual consultation where there is no provision for physical examination, which is an integral part of physician–patient consultation. It also shows the importance of requesting appropriate investigations to make up for the lack of physical assessment. Referring to the right specialty and switching the mode of consultation should also be considered when deemed necessary.

**Figure 5:**
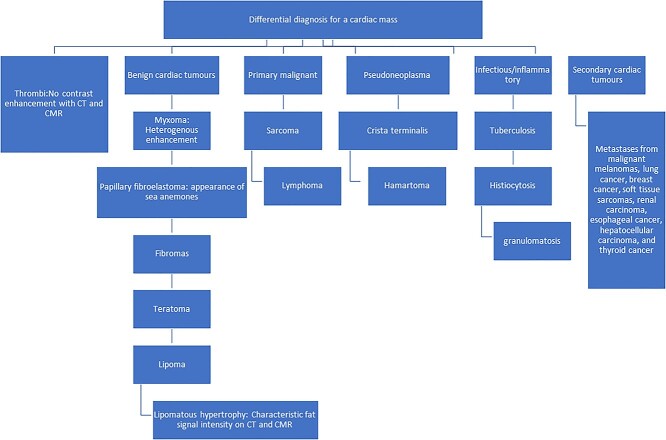
DD of atrial myxomas.

Cardiac myxomas are more common in women and present with constitutional symptoms [1] such as poor appetite, marked fatiguability and weight loss, which might be the only presentation. Therefore, for the prevention of catastrophic complications from embolization or heart failure, a systematic history and examination leading to early diagnosis are paramount [2]. RA masses produce haemodynamic changes that are similar to those seen with right heart failure. A diastolic murmur has been described on physical examination, which is similar to the ‘tumour plop’ heard with left AM. Ninety per cent of the myxomas arise sporadically, whereas 10% are familial [3], arising from multipotent mesenchymal cells [4]. The attachment point can be broad, sessile, narrow or pedunculated [5] and they are macroscopically gelatinous with smooth, villous, friable surface, which can embolize in 35%. Histologically, myxomas have a myxoid matrix with embedded myxoma cells, pigmented macrophages, haemosiderin and intratumoural calcifications. The main DD for a myxoma is thrombus [5] due to its heterogeneous appearance and confirmation is required, as anticoagulation is not proven to reduce embolic events in myxoma. Figure 5 shows other DDs that are worth considering.

As seen in our patient there is a diagnostic uncertainty with TTE, which is seen with sessile tumours with no interatrial septal (IAS) attachment, history of cancer and atypical presentations [5]. Advances in TTE such as quantitative perfusion and 3D imaging help in differentiating perfusion patterns between malignancies (hyper-enhancing pattern), benign stromal tumours (hypo-enhancing pattern) and thrombus (non-enhancing pattern) [5]. TOE is superior to TTE especially for myxoma detection (100 vs. 95%) and attachment point identification (95.2 vs. 64.5%) [5]. Our patient had a TOE which confirmed an atrial mass but with a DD of myxoma or thrombus. She then had a CT scan and CMRI followed by an MDT before the diagnosis of AM was made and surgery was planned. If the diagnosis is not definite, further characterization using CMRI [5] with contrast-enhanced sequences and late gadolinium enhancement is important in distinguishing myxomas from thrombus [6]. Coronary angiography is important in mapping the vascular supply of tumours arising from epicardial arteries as they may require resection and grafting [1]. Table 1 shows different imaging modalities, their characteristics, advantages and limitations in diagnosing AMs.

**Table 1:** Different imaging modalities with their advantages and limitations in diagnosing AMs

Echocardiography	Transesophageal echocardiography	CMR	Gated cardiac CT	PET
• Most widely available, cost effective and noninvasive first-line imaging. Locates the mass, size, obstructive and embolic features [3] • limited due to poor acoustic windows	• Close proximity of the oesophagus to the heart [8], good resolution providing better quality images. • Invasive examination, at times not tolerated well.	• T1-, T2-weighted sequences and cine imaging has superior tissue characterization and resolution [3]. • Occurrence of artefacts, difficulty in detecting small mobile masses [4]. • Cannot be used in the presence of contraindications for the use of magnetic resonance imaging.	• Can be used to exclude a cancer from fat or calcifications [4]. • precontrast and post-contrast images may not be sufficient to differentiate myxomas from thrombi.	• A PET scan can be used to differentiate myxoma from metastatic involvement [3, 8] • However most myxomas show no or low uptake [4].

Cardiac imaging helps with patient selection for surgical removal, anticoagulant therapy, oncologic therapy or conservative management. Characterization of the tumour is important as surgical complications vary depending on the diagnosis [7, 8]. Results from surgical resection are good, with <5% mortality rate. Postoperative recovery can be complicated with arrhythmias reported in a few patients. There is a recurrence rate of 2–5% most commonly in patients whose primary tumour was multicentric [1].

## CONCLUSION

As we continue to use telemedicine during the COVID-19 pandemic to minimize the spread of infection along with reducing the total cost to the health care system [9], this case report highlights the importance of a systematic history, recognizing red flags and appropriate imaging in the early diagnosis and management of conditions that can cause life-threatening complications.
